# State Medicine and Public Hygiene

**Published:** 1877-07

**Authors:** J. Suydam Knox


### SECTION V.—STATE MEDICINE AND PUBLIC HYGIENE.

Reported by J. SUYDAM KNOX, M. D.

FIRST DAY—TUESDAY, JUNE 5TH.

Dr. E. M. Hunt, N. J., Chairman; Dr. Waterman, Ind., Secretary pro tem.

After a brief introductory address by the chairman, and the transaction of some miscellaneous business, Dr. J. L. Cabell, of Va., was introduced, and read an able and exhaustive paper upon the Etiology of Enteric Fever.

The germ theory of disease was reviewed; the different views of Budd and Murchison commented upon; and the arbitrary manner of English writers in limiting the causation of enteric fever to one source criticised.

In order to study the etiology of enteric fever, the writer addressed a series of questions to physicians of eminence in different parts of the Union, especially the State of Virginia. Many of the replies were contradictory. After defining the difference between direct and miasmatic contagion, positive cases and opinions were quoted from these replies, for and against direct contagion. The conclusion reached was, that while the evidence of direct contagion was often incomplete, there was positive proof of contagion from miasmatic influences; viz., infected water or air. Many positive opinions were also quoted of the *de novo* origin of the disease, and the cases cited. Still, against such an origin it may be said that the disease may occur under favorable conditions, where direct importation may be excluded; thus from germs left long ago, or from germs latent in the body and developed by favorable circumstances, or by wide diffusion in the air, of dessicated but living germs.

As a summary from the answers received, the following may be stated as external conditions favorable to the development of enteric fever:

1. Excremental filth undergoing moist decomposition, and contaminating air and water.
2. Contaminated milk.
3. Vegetable decomposition.
4. Decay of dry timber.
5. Soil saturated with organic impurities independently of water contamination.
6. Contaminated water.
7. Some undefined telluric influence not associated with organic contamination.

In regard to the relations between typhoid and malarial fevers, a majority of the correspondents consider them distinct and of separate origin. A few recognized hybrids. Most consider typho-malarial fever to be an adynamic form of malarial fever, due to pythogenic influences. Some recognize a decided antagonism between the two, and quote the marked absence of typhoid in malarial regions, and if the former does occur it is of mild type. The drying up of a marsh producing malaria, will introduce a new type of fever; viz., typhoïd. Abundant illustration of this is seen in Illinois and the South. May be due to the fact that malaria requires a saturated soil, and typhoid a dessicated one.

English theories exclude epidemic influences, yet apparently typhoid fever may extend in the track of atmospheric currents independent of direct intercourse. Indeed, the observation of some gives it a migratory character, due neither to local causes nor yet to direct contagion. Further observation is necessary to establish many of the above conclusions. To do this, isolated cases must be studied, and meteorological conditions during the presence of an epidemic must be observed.

In the discussion that followed, Dr. Comyges, of Cincinnati, reported a succession of cases in his own family, proving the infectious character of enteric fever.

Dr. Plummer, of Rock Island, insisted that typhoid and malarial fever can occur at the same time in the same patient. Proven by post-mortem examinations. There were many such specimens in the Washington Museum.

Dr. Woodward, U. S. A. Wished to emphasize the fact that typhoid and malarial fevers alternate, and also another fact, that sometimes on a colossal scale, they occur together. This was largely illustrated in the Union army during the late war. Another point he wished to make, was that men, who deny the existence of typho-malarial fever do not make post-mortems. When examination is made of fatal remittants, enteric lesions are found. Again, the only facts supporting the germ theory of disease, are those resulting from inoculations. What is the material inoculated? Plainly diseased tissues and germs combined. Which produces the resulting disease, the tissue or the germs? Logically, the diseased tissue, rather than the germs, since all diseased tissues differ, whereas germs, chemically, morphologically and microscopically, are the same.

Dr. Pratt, of Michigan, spoke positively, from experience, of the modifying influence of malaria upon typhoid. During a practice of 20 years, in a malarial district, he had never seen there a case of enteric fever.

Dr. Freeman, of Missouri, had frequently, in his state, seen malarial fever driven out by typhoid, and vice versa, and also to co-exist with it. He had seen desperate cases of typhoid much benefited by being taken into malarial districts.

Dr. Hoar, or Maryland, made remarks of similar import.

Dr. English, of New Jersey, called for the publication of Dr. Cabell's paper, which was moved and passed.

### Second Day, Wednesday, p. m., June 6th.

Dr. J. R. Blach, of Ohio, opened the proceedings with a paper on "The Laws of Heredity, with Special Reference to the Transmission of Morbid Tendencies, Abnormal Forms, and the Effects of Intermarriages." He said that the improvement of the domestic animals had long been the source of earnest thought and care, and the objects sought to be accomplished, whether beauty, strength, or endurance, they had been eminently successful in obtaining. As regards the human race, nothing of the kind had been attempted, everything being left to chance except as regards efforts to train the head and heart. The leading object of this culture was social success, and to gain this the welfare of blood and brain was often sacrificed. There was no wonder that the boasted head of creation should be at its foot so far as vigor, health, and longevity was concerned. It was certain that the stamina of man as a race was, if not retrograding, at least not advancing. The especial reason of this was the over-exercise of some and the enforced inaction of other organs. Brain-toil was commenced far too early, ere yet the brain was capable of sustaining it, and only the strongest young men and women were able to go through a long collegiate curriculum unscathed by disease. This fact, demonstrated before the fertile period had been reached, had a special bearing upon hereditary modification. During the more primitive forms of civilization, weak and imperfectly developed children were more likely to perish by the hardships to which they were exposed. Physical strength was more sought after in those days, out-door exercise was general, and the diet simple and wholesome. To-day every possible means were adopted to keep such children alive, and when they reached manhood or womanhood they were advised and encouraged to marry healthy persons, thus aiding to extend and intensify the prevailing blood-deterioration. The consequence was the multiplication, to an alarming extent, of hospitals and infirmaries.

The inference of degeneration was shown by vital statistics. In Rhode Island, for instance, the birth-rate had sunk lower than in any European nation with the exception of France. It was found, and would be found, necessary to multiply hospitals and infirmaries if the laws of health were to be ignored. It would seem, if this was to be kept up, that, as the process went on, one-half of a nation would be kept in constant employment in caring for the helpless and diseased other half.

The speaker had addressed 250 circulars to leading physicians, asking their personal experience as to the transmission of hereditary infirmities, and tendencies to disease, and to the effects of inter-marriage. Sixty-five responses were received, embracing the vital history of ninety-three persons. Of these only thirty-three, fifteen males and eighteen females, were reported as free entirely from inherited predisposition to disease. The remarkable predisposition through reproduction of some sexual variation of a normal character was indisputable, but it was not proved that the same law extended to abnormal variations. It had been observed that the Jewish race was more free from disease and longer-lived than other races in the same environment. There was a marked tendency to reversion to older or more durable types, especially when the modifying influences were withdrawn. All authorities agreed that characteristics became more firmly fixed as they were transmitted through generations. The many varieties of pigeons which breeders had been able to produce by careful selection, one and all, when left to natural influences, reverted to the primitive type. This was also shown in regard to cases of congenital defects. When a male or female deaf-mute married a sound person the offspring was very seldom affected. The result of crossing varieties of domestic animals was often a reversion to the primitive type.

Through the reports received from some of the most advanced medical thinkers of the day, many typical instances of recovery or reversion from an inherited tendency to disease, when the conditions favorable thereto had been faithfully and skillfully carried out, had been collated. Reversion from acute diseases was an every-day affair. A large number of instances of the reproduction of congenital peculiarities and abnormal forms were cited by the lecturer, who then proceeded to give some particulars as to

CONSANGUINE MARRIAGES, the statistics being derived in the same manner and relating entirely to physicians or their connections. In twenty of these the degree of relationship was cousin-german, with the following results upon their offspring: No ill effects in five cases; in the sixth, three of the children had supernumerary fingers and toes; the seventh, eighth, ninth, tenth, eleventh and twelfth, one or more children imbecile or idiotic, two nearly blind and deaf, one a dwarf, one epileptic. In the thirteenth instance, one had a cleft palate; in the fourteenth, two were strikingly handsome, but below par in intellect; in the fifteenth, one sickly and feeble; in the sixteenth, one without the right arm but with rudimentary fingers on the shoulder; in the eighteenth, one had talipes equinus; in the nineteenth, all the progeny were unhealthy and scrofulous; and in the twentieth instance, of a large family, all were hideously deformed, though clear and bright in intellect. From these figures, the deduction was made that the chances of the issue of consanguineous marriages, having well-formed bodies and sound minds, were so small that the strongest legal enactments against them were justified.

In conclusion, the lecturer declared that the more the primitive phases of civilization were considered, the more apparent did the fact become that the somewhat violent hardships and privations to which life was then exposed, fell with special force on the weak and helpless, and little, if at all, on the vigorous, thus tending to the elimination of the one and the perpetuation of the other. It was apparent, further, that few persons possessed the strength of mind necessary to abstain from the perpetuation of the species, simply because they inherited a congenital disorder. Yet even this fact had an outcome not to be deplored. High intelligence, strong wills, and consistent behavior would survive, while feeble-minded ignorance and volitions unstable as water would carry the blood on to imperfection, disease and extinction.

Dr. A. N. Bell, editor of *The Sanitarian*, of New York, followed with an exhaustive article on "Tuberculosis in milch cows, and the contagiousness of tuberculosis of the digestive organs." This was one of the most valuable papers read before the American Medical Association this year. It contains an account of Dr. Bell's personal investigations and observations carried on in the various veterinary schools of Europe. The statistics from numerous observers in the various civilized countries were carefully collated and compared. The writer displayed great industry and patience in preparing his paper, and the conclusion to which his investigations and those of other original workers and observers led, is the certain transmissibility of tubercles, or of the tubercular dyscrasia to eaters of meat from animals affected with *tubercle.*

Dr. Bell pointed out the exceedingly great importance of securing the services of comepetent meat inspectors, and of enforcing a police espionage over our meat stalls, for sanitary purposes.

Dr. Comegys, of Ohio, then read a paper containing resolutions which are herewith appended. He contended for a more rigid enforcement of the laws in regard to the practice of medicine. Unqualified persons should not be allowed to practice, and the State should exercise a careful supervision over the issuance of diplomas. Another point was that the medical profession was practically excluded from political life. The lecturer concluded by offering the following resolutions, which he respectfully submitted for the consideration of the Committee on State Medicine:

*Resolved*, That the medical profession, by reason of its paramount claims as guardian in so great a degree of the best interests of society, should seek to give authority to its claim by making the State the expression of it.

*Resolved*, That it is the duty of the medical profession to labor for the establishment of a Medical Council of State in each State, which shall be empowered by law to make rules and regulations for the qualifications of medical practitioners, and the regulation of whatever is connected with public hygiene.

*Resolved*, That it is the duty of the profession to exert its influence to secure a fair representation of physicians in legislative bodies, because their participation in legislation is most important to the public weal.

*Resolved*, That the devotion of physicians to medical politics will not hinder their culture or usefulness as practitioners, but rather will greatly strengthen them in their ability to promote good order and human happiness.

The Chairman then announced that discussion was in order, and Drs. Bell, of New York, Pratt, of Kalamazoo, Mich., and Lyster, of Michigan, made a few comments on Dr. Black's paper, which was referred to the Committee on Publication. Dr. Bell's paper was also so referred with the proviso that it might be taken up for discussion at a future meeting.

THIRD DAY, THURSDAY, JUNE 7.

Dr. Elisha Harris, of New York, presented a paper upon "Results of State Legislation on Public Health." Owing to other engagements, Dr. Harris was unable to read his paper on this subject, but after submitting the paper, presented the following suggestions as subjects of discussion:

1. In organizing boards of health, the manner of conferring authority on men already over-burdened with other duties, fails to give sufficient authority or to secure the confidence of the people.

2. In defining the powers and duties of local boards of health, the powers conferred are too indefinitely stated, and the laws of nearly all the States, upon this subject, need revision.

3. The proper definition of nuisances, often answers the purpose of abating them.

4. There is need of specific provisions against contagious diseases.

Such laws as exist have come to be regarded very much misunderstood and abused, so as to bring into disrepute medical boards.

A broader basis must be secured through conference with army, navy, and state medical boards.

Thus there should be a national basis for laws of quarantine.

There should also be general regulation of certain sources of contamination of certain articles of food.

We know that one of the sources of impairment of animal food, is due to want of sanitary care of animals in transportation. This is a worthy subject, and requires proper legislation.

Also the subject of trichinæ requires scientific observation and treatment.

5. Laws are required for the proper removal and prevention of general sources of diseases, such as sewerages and sanitary drainage.

Something more than the dicta of local boards of health or local engineers, is needed. This question involves the health and wealth of States as much as certain localities, therefore there is needed general and systematic topographical state surveys.

After a general discussion of the subjects suggested the business of this section closed.

